# Molecular weight of surface immobilized hyaluronic acid influences CD44-mediated binding of gastric cancer cells

**DOI:** 10.1038/s41598-018-34445-0

**Published:** 2018-10-30

**Authors:** Sara Amorim, Diana Soares da Costa, Daniela Freitas, Celso A. Reis, Rui L. Reis, Iva Pashkuleva, Ricardo A. Pires

**Affiliations:** 1 3B’s Research Group, I3Bs – Research Institute on Biomaterials, Biodegradables and Biomimetics, University of Minho, Headquarters of the European Institute of Excellence on Tissue Engineering and Regenerative Medicine, AvePark, Parque de Ciência e Tecnologia, Zona Industrial da Gandra, 4805-017 Barco, Guimarães Portugal; 2 ICVS/3B’s - PT Government Associate Laboratory, Braga/Guimarães, Portugal; 3 The Discoveries Centre for Regenerative and Precision Medicine, Headquarters at University of Minho, Avepark, 4805-017 Barco, Guimarães Portugal; 4 Instituto de Investigação e Inovação em Saúde - i3S, Universidade do Porto, Porto, Portugal; 5 Institute of Molecular Pathology and Immunology of the University of Porto – IPATIMUP, Porto, Portugal; 6 Institute of Biomedical Sciences Abel Salazar, University of Porto, Porto, Portugal; 7 Department of Pathology and Oncology, Faculty of Medicine, Porto University, Porto, Portugal

## Abstract

The physiological importance of the interactions between hyaluronic acid (HA) and its main membrane receptor, CD44, in pathological processes, e.g. cancer, is well recognized. However, these interactions are mainly studied in solution, whereas HA in the extracellular matrix (ECM) is partially immobilized via its interactions with other ECM components. We therefore, developed substrates in which HA is presented in an ECM-relevant manner. We immobilized HA with different molecular weights (M_w_) in a Layer-by-Layer (LbL) fashion and studied the interactions of the substrates with CD44 and two human gastric cancer cell lines that overexpress this receptor, namely AGS and MKN45. We demonstrate that MKN45 cells are more sensitive to the LbL substrates as compared with AGS. This difference is due to different CD44 expression: while CD44 is detected mainly in the cytoplasm of AGS, MKN45 express CD44 predominantly at the cell membrane where it is involved in the recognition and binding of HA. The invasiveness of the studied cell lines was also evaluated as a function of HA M_w_. Invasive profile characterized by low cell adhesion, high cell motility, high expression of cortactin, formation of invadopodia and cell clusters was observed for MKN45 cells when they are in contact with substrates presenting HA of high M_w_.

## Introduction

Hyaluronic acid (HA) is a non-sulfated glycosaminoglycan (GAG) that is present in the extracellular matrix (ECM) of all mammalian cells^1^. It is involved in key signaling pathways determining cell fate but also progression of some malignant tumors^1–4^. One of the main transmitters of the HA bioactivity is the transmembrane glycoprotein CD44 – the principal cell surface receptor of HA^1–3,5,6^. CD44 can be expressed as different isoforms: the most common standard isoform, CD44s, is expressed in most cells, while some CD44v (CD44 variants containing variable exons) are associated with specific tumors^5–8^. In fact, the CD44+ subpopulation of cancer stem cells (CSCs) in solid tumors has been identified as cancer-initiating cells^9^. This subpopulation is resistant to chemo- and radio-therapies commonly employed in cancer treatment^10^. Under specific culture conditions, cancer cell lines, such as AGS and MKN45, behave as CSCs, i.e. they express CSC markers (e.g. CD44) and have self-renewal capacity, present high proliferation rate, and exhibit high drug resistance, among other characteristics^11^.

Together with CD44, endogenous HA is also involved in different stages of malignant tumor progression. HA expression is usually up-regulated in the tumor microenvironment^1,3,4^. The interactions between HA and the membrane receptors of cancer cells activate several signaling pathways that promote tumor cell growth, survival, and migration, thereby increasing metastatic spread^1,3,4^. The concentration of HA and its M_w_ is of crucial importance for these interactions. In humans, cells secrete HAs with different M_w_s in ECM: HAs of high M_w_ (up to 2 MDa) are generated by hyaluronan synthases HAS1 and HAS2, while the analogues with lower M_w_ (up to 200 kDa) are produced by HAS3^12^. When secreted by the cells, these HAs can be further modified by hyaluronidases (HYALs) – specific enzyme family that catalyze the degradation of HA – to generate species with even lower M_w_, including the oligo-HAs that present M_w_s of up to 6.4 kDa (16 disaccharide units)^13^. The delicate balance between synthases and hyaluronidases determines the HAs composition in the ECM and depends on the specific cells/tissues and their pathological state. Generally, HAs with high M_w_ (M_w_ > 500 kDa) are space-filling molecules involved in normal biological processes. They have anti-angiogenic and immunosuppressive properties. Usually, they are associated with inhibition of cellular differentiation by suppressing cell–cell interactions or ligand access to cell surface receptors. In contrast, the shorter HA fragments (M_w_ = 20–200 kDa) have pro-inflammatory, immuno-stimulatory and angiogenic properties. HAs with low M_w_ are detected in cancers. Their effect is contradictable: some studies relate low M_w_ HA with enhanced cancer cell invasion^14–16^, while others report reduced invasiveness in the presence of these HAs^17^. These contradictions can be explained with the used models to study this effect and their relevance to the *in vivo* scenario.

Different approaches have been developed to elucidate the HA-CD44 interactions^18,19^. Of note, most of these approaches focus on interactions occurring between molecules in solutions, and there is limited information regarding the interactions between immobilized HA and CD44 and/or cancer cells. The presentation of HA in such context of spatial confinement, with restricted flexibility, is of great interest in the design and development of ECM mimics and biomedical devices, e.g. biosensors, in which functionalization with HA is required.

In the present work, we report on the interactions of immobilized exogenous HA of different M_w_s (oligo, 6.4 kDa; medium, 752 kDa; and high, 1500 kDa) with two gastric cancer cell lines (AGS and MKN45). We applied the Layer-by-Layer (LbL) approach to deposit HA of different M_w_s on tissue culture polystyrene (TCPS). This approach allows surface immobilization of biomolecules with minimal or no changes of their chemistry, i.e. with preserved bioactivity. Moreover, the immobilized molecules are presented in an ECM fashion: they are spatially restricted but enough flexible to reorganize and bind CD44.

## Results and Discussion

### Immobilization of HA

**Figure 1:**
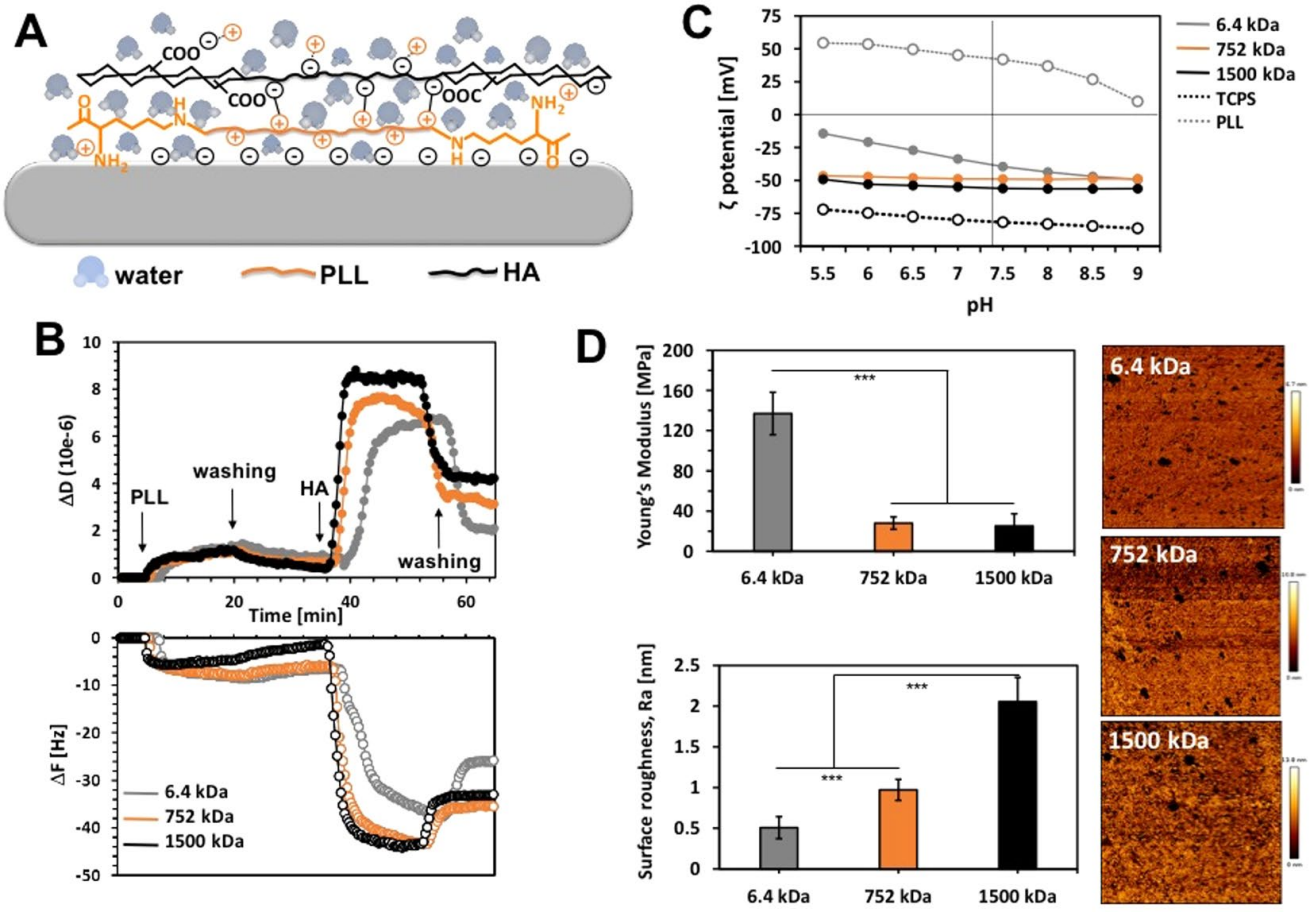
Schematic presentation of the used substrates (**A**) and their characterization by QCM-D (**B**), electrokinetic analysis (**C**) and AFM (**D**). All AFM data were obtained after scanning areas of 150 × 150 nm. The significant differences are marked by (***) for *p* < 0.001.

Biomacromolecules can be immobilized on different substrates following two general approaches. In the first one, a covalent bond between the substrates and the biomolecule is formed. This approach generates stable and chemically defined platforms but usually requires functionalization of the support and the biomolecule – a process that can compromise the bioactivity of the immobilized component either by modifying its active centers or by restricting its mobility, i.e. ability of binding sites alignment^20–22^. On the other hand, non-covalent immobilization does not require any pre-functionalization of the biomacromolecules. It relies on their simple deposition (usually by physical adsorption) on properly modified substrates. These approaches are associated with higher flexibility of the deposited molecules but also with a limited stability: while polymers with high M_w_ assemble in relatively stable coatings, their oligo-derivatives form unstable surfaces^21^. Herein, we applied the LbL approach to immobilize and present HA in an ECM resembling manner. The most common LbL assembly relies on alternate deposition of polyelectrolytes with opposite charges^23,24^. We therefore used HA (a weak polyanion) in combination with poly-L-lysine (PLL, strong polycation). This system is well studied by us and others: it has been shown that the M_w_ of the polyions can alter dramatically both the structure of the multilayer system and its stability^25–30^. Based on these previous findings and the requirement to test HA with different M_w_, we have used a bilayer system that was crosslinked via common carbodiimide chemistry. We expected that such constructs would be stable and with limited “in and out” mobility, i.e. the layers would be defined, and that HA present on the surface of the construct would be enough flexible to reorganize and bind CD44 (Fig. 1A).

The deposition of the layers was followed *in situ* by quartz crystal microbalance with dissipation (QCM-D, Fig. 1B). This acoustic technique is based on a quartz crystal disk (QCM-D sensor) that oscillates at its resonance frequency when an alternating potential is applied. The resonance frequency (F) changes upon mass adsorption on the surface and the deposited mass can be calculated from the Sauerbrey’s equation when rigid films are formed. Beyond the Sauerbrey limit, the viscoelastic properties of the films can be accessed using an appropriate model such as the Voigt one^31^. Besides the deposited mass, QCM-D also allows quantification of the viscoelastic properties of the deposited films. Such measurements are performed by continuous switch on and off of the driving potential that result in a decay of the freely oscillating crystal and allows measurement of the energy dissipation (D).

The adsorption of PLL and HA was confirmed by a decrease of F and an increase of D (Fig. 1B). The changes were more pronounced during the build-up of the HA layer (regardless the M_w_) than during the PLL deposition. These results are in a very good agreement with previous reports that refer to the high hydration state of the HA as a main reason behind this difference^25,26,30^. In fact, the AFM characterization confirmed this explanation (Fig. 1D): the deposition of the first PLL layer resulted in a formation of relatively stiff surface (~600 MPa, data not shown). The following adsorption of the HA led to a sharp decrease of the Young’s Modulus that is a result from the high capacity of HA to adsorb and retain water, i.e. to form very swelled films^32^. This difference was much bigger for the HA with medium and high M_w_ (~25 MPa for 752 and 1500 kDa) than for the oligo-HA (~140 MPa for HA of 6.4 kDa) and it is consistent with previous reports^27,32,33^. The results obtained for the roughness (Ra) of the substrates are also in agreement with the literature. We observed formation of “islets” upon adsorption of the HA (brighter areas in Fig. 1D)^30,33^. The size of these islets and the Ra of respective bilayer depends on the M_w_ of the HA - larger islets are formed from HA with higher M_w_.

The preservation of the LbL structure, i.e. lack of layers’ migration and the presence of the HA on the substrate’s surface, was confirmed by electrokinetic analysis (Fig. 1C). This characterization method gives information about the ζ-potential of the surface. The deposition of PLL onto TCPS altered the ζ-potential, from negative to positive: at pH 7.4 (physiological pH) the surface charge of the substrate changed from −82 mV to +42 mV. The following deposition of HA led to a reversion of the ζ-potential to negative values. Of note, the ζ-potentials of the surfaces presenting HA of 752 and 1500 kDa do not change significantly with the pH, indicating the formation of stable and chemically defined constructs. The bilayers formed from oligo-HA, i.e. 6.4 kDa, present similar behavior at basic and neutral pHs. However, at acidic pH we observed an increasing ζ-potential for this system, indicating instability or possible layer migration at these pH values^27^.

### Interactions of LbL with CD44

**Figure 2:**
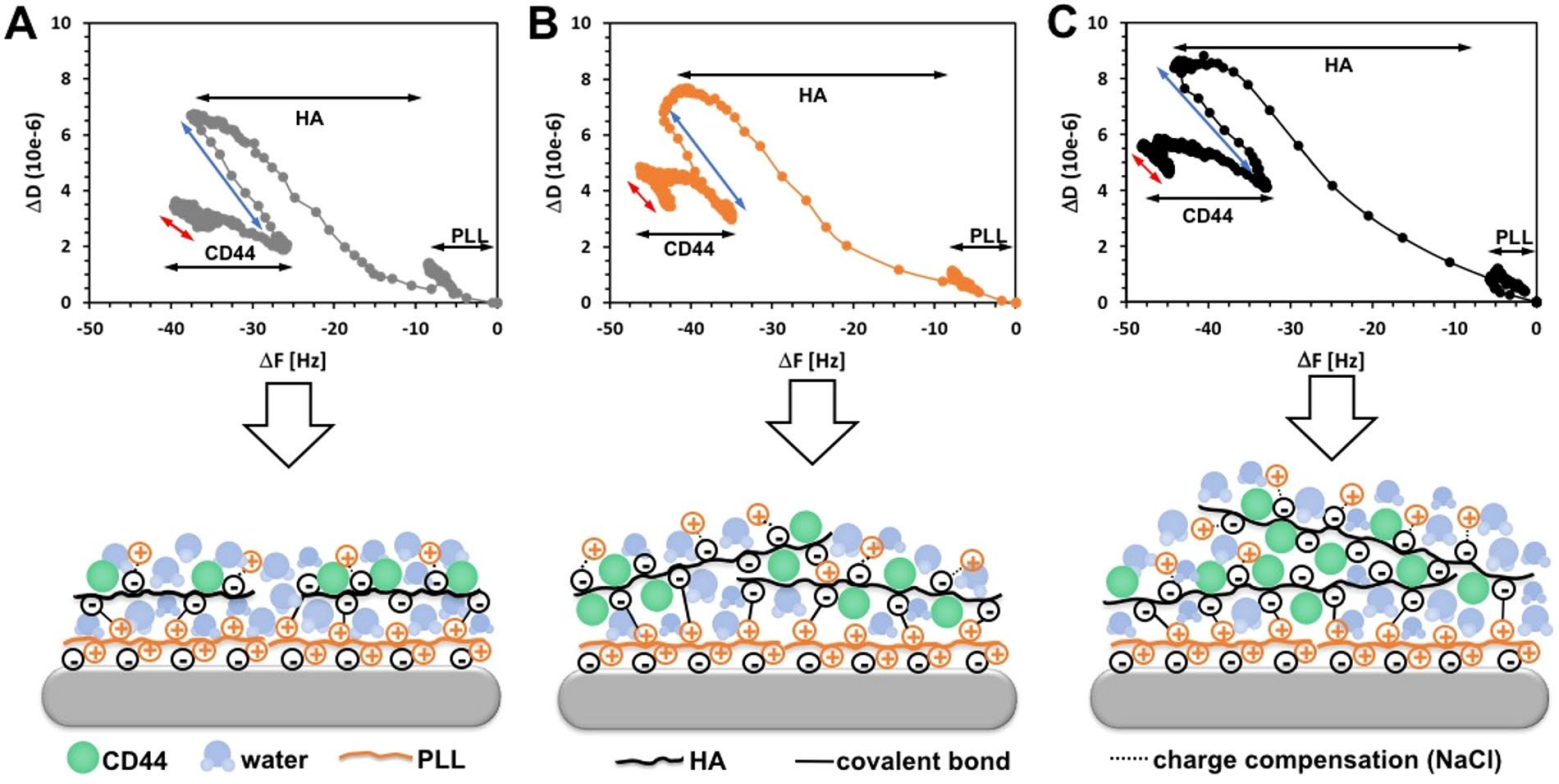
D/F plots and respective models for the interactions between CD44 and the surfaces prepared with HA of 6.4 kDa (**A**), 752 kDa (**B)** and 1500 kDa (**C**). The arrows indicate each layer build-up (black) and the respective washing steps after HA (blue) and CD44 (red) depositions.

The M_w_ and presentation of HA (soluble or immobilized) affect tremendously the activation of its receptors, the binding to these receptors and the resulting downstream signaling^15,16,34,35^. The most widely accepted theory suggests that the HA-receptor binding relies on multivalent interactions and the downstream signaling is related with the ability of HA to cluster the receptors on the cell surface (Supplementary Fig. S1). The minimal binding size of HA depends on the receptor: 6-mer of HA binds to CD44, while 4-mer does not interact with this receptor^2,15^. Importantly, 6- to 18-mers of HA bind CD44 monovalently and thus, impede the following clustering and signaling. Size-dependent HA signaling can also differ according to cell type and HA presentation. So far, most of the generated results consider HA in solution, usually as a supplement to the cell culture media. However, *in vivo* HA is embedded in the ECM, where it interacts with other bioentities that restricts HA spatial freedom and defines spatially its binding sites^16^. The LbL substrates designed by us are reductionist model of this environment. We characterized their interactions with CD44 *in situ* using QCM-D (Fig. 2).

Herein, we present the QCM-D data as D/F plots because this kind of presentation visualize readily the dissipation caused by a unit of frequency (mass)^36,37^. The results clearly demonstrate that CD44 interacts with all LbLs. While this technique does not allow quantification of the retained protein (because the signal change includes also the water retained in the LbL), it is interesting to see differences among the studied substrates. The CD44 deposition on the surface presenting HA of 6.4 kDa resulted in a decrease of F and an increase of D, i.e. in a linear change of D/F plots (Fig. 2A), indicating formation of a protein monolayer on the top of the constructs. Different signal shape is observed for the LbL formed from HA with medium and high M_w_s (752 and 1500 kDa). The initial CD44 deposition follows the same trend as for the surface presenting HA of 6.4 kDa but then, we observe deposition of mass (decrease of F) that is not accompanied with changes of D (Fig. 2B,C, the plateau of the CD44 part in D/F plots). This signal indicates that after initial deposition on the substrate surface, CD44 penetrate within the constructs where it replaces the water (the schemes in Fig. 2B,C). This process is only valid for longer HA chains that form LbL with more free volume in their vicinity (as shown by the QCM-D data in Fig. 2: grey line vs orange and black ones in ΔD plot). Moreover, the degree of freedom of these HA chains is higher and they are able to reorganize better and capture the CD44 from the solution.

### Cell adhesion and morphology of AGS and MKN45 seeded on LbL

We studied the behavior of two different gastric cancer cells, namely AGS (intestinal type of gastric carcinoma) and MKN45 (diffuse type of gastric carcinoma). According to previous reports, these cell lines overexpress CD44s and some CD44v, e.g. CD44v6, which are associated with cancer^38,39^. Patients with gastric carcinoma and high expression of CD44v6+ are usually diagnosed with advanced stage of the disease, related with high metastasis potential and mortality^10,40–43^.

Our results confirm that indeed both cell lines express CD44 when cultured on TCPS, although there are differences among them: while all MKN45 are CD44s+ and very big population is CD44v6+, only about half of AGS cells are CD44s+ and CD44v6+ (Table 1 and Supplementary Fig. S3)^10^. Besides larger CD44+ population, MKN45 cells express much higher amount of CD44s as demonstrated by the mean fluorescence intensity (MFI, Table 1).

**Table 1:** Expression of CD44s and CD44v6 in AGS and MKN45 cells as a function of the substrate surface composition. The quantification was performed by flow cytometry after 72 h of cell culture in contact with the respective substrates.

	AGS				MKN45			
	TCPS	HA6.4	HA752	HA1500	TCPS	HA6.4	HA752	HA1500
	**CD44** ^**+**^ **cell populations [% from the total cell population]**							
CD44s	47.08	85.07	94.10	94.96	99.08	94.81	95.14	95.13
CD44v6	50.30	77.37	57.14	56.25	83.11	63.30	73.94	76.69
	**Mean Fluorescence Intensity (MFI)**							
CD44s	13.41	20.61	30.71	31.23	193.51	164.25	137.37	140.91
CD44v6	14.12	16.50	14.96	14.41	20.57	18.76	19.36	22.51

**Figure 3:**
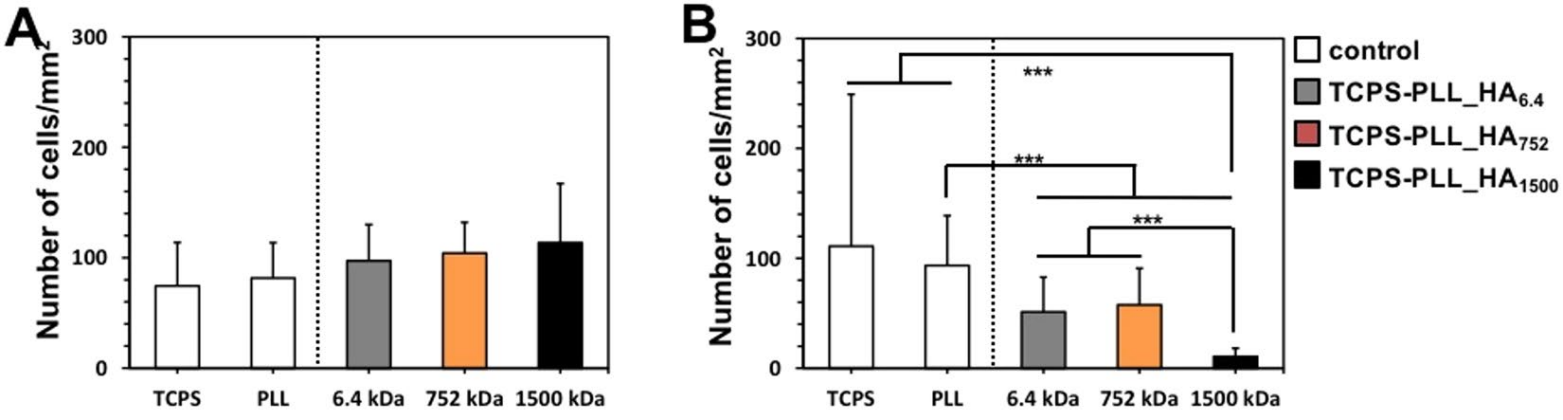
Number of adherent AGS (**A)** and MKN45 (**B**) cells after 72 h of culture on different substrates. Significant differences are marked with ***(*p* < 0.001).

In the absence of HA, i.e. on TCPS and on PLL substrates, we observed comparable number of adherent AGS and MKN45 cells (Fig. 3 white bars). Upon addition of HA to the cell culture medium, we did not observe any effect of the M_w_ of the supplemented HA either for AGS or for MKN45 cells (Supplementary Fig. S2). When cells were cultured on the LbL constructs with HA, the number of the adhered AGS cells did not change significant (about 100 cells/mm^2^, Fig. 3A), while less attached MKN45 cells (maximum 58 cells/mm^2^) were determined on these substrates and this difference was most significant for the surface presenting HA of 1500 kDa. Possible explanations of this result are the different expression and/or availability of the CD44 (CD44s and CD44v) on the surface of the studied cell lines in response to the substrate change or/and different adhesion mechanism of AGS and MKN45 cells. We, therefore, used flow cytometry to quantify the percentage of cells that expresses CD44s and the CD44v6 (positive cell population) and the quantity of the protein they express on their surface (Mean Fluorescence Intensity, MFI) in contact with the surfaces presenting HA of different M_w_s (Table 1 and Supplementary Fig. S3).

The presence of HA on the substrates induces different response of the studied cells. AGS cells recruit CD44s on their surface: bigger CD44+ populations and higher expression (MFI, Table 1) of CD44s were determined for these cells in contact with LbL as compared with cells cultured on TCPS. While the trend is the same for all LbL substrates, cells cultured on LbL of the oligomeric HA (6.4 kDa) show smaller increase in terms of both population size and MFI. The substrate change affect less the expression of CD44v6 in AGS but in this case the LbL of shortest HA analogue induces the bigger change. Opposite effect was observed for MKN45 cells. The CD44s(+) population is preserved but the cells express less protein on the surfaces (smaller MFI as compared with the TCPS one) in contact with LbL and this effect was less pronounced for the substrates with HA of 6.4 kDa (Table 1). The presence of HA affect also the expression of CD44v6 by MKN45: smaller CD44v6(+) populations were determined in this case.

**Figure 4:**
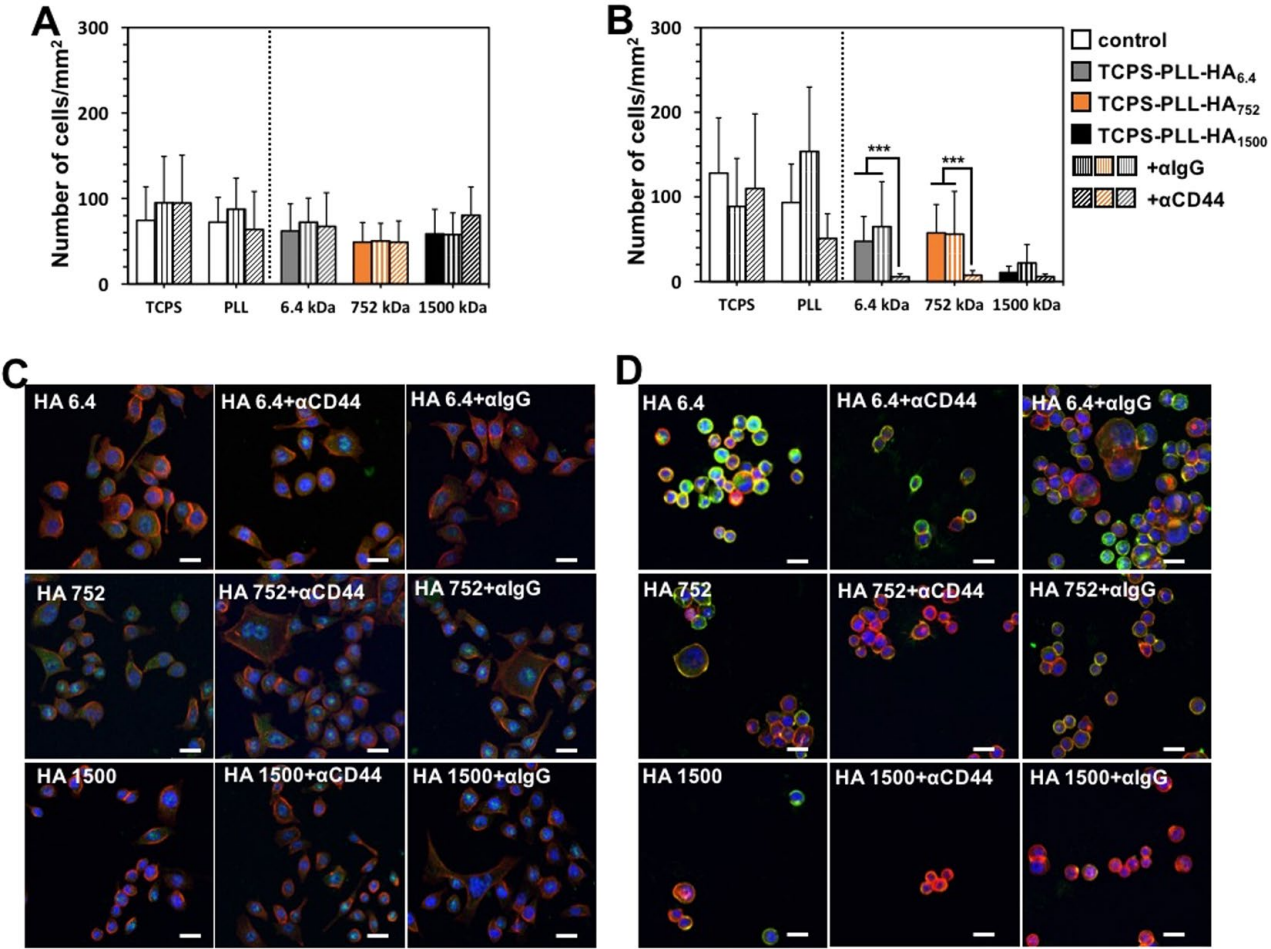
Effect of CD44 blocking on the adhesion of AGS (**A**,**C**) and MKN45 (**B**,**D**) presented as number of adherent cells (**A**,**B**) and visualized by immunohistochemistry (green for CD44, red for actin and blue for nuclei). The results were obtained for cell cultured during 78 h. Bars correspond to 50 µm. IgG was used as an isotype control. Significant differences (Kruskal-Wallis, Mann-Whitney test, *p* < 0.001) are marked with ***. Images for the control substrates (TCPS and TCPS-PLL) are presented in Supplementary Fig. S4.

The involvement of these receptors in the adhesion to the LbL was evaluated by blocking them and studied the behavior of AGS and MKN45 in contact with the same substrates (Fig. 4). The CD44 blocking did not affect AGS attachment (Fig. 4A) but significantly less MKN45 cells were observed on all HA functionalized substrates (Fig. 4B) indicating that MKN45 cells adhesion is CD44-mediated. AGS cells used other mechanisms of adhesion, which do not involve CD44-HA interactions, possibly through integrin mediation^44,45^. These seemingly contradictory results for AGS cells (recruiting CD44 but not using them for HA binding) can be explained by structural differences of the CD44 expressed by these cells. Differences in the M_w_ (different variant isoforms generated by alternative splicing) and glycosylation profile can affect tremendously the HA binding^39,46^. Usually, poor HA binding is related with steric hindrance of the binding sites caused by extensive CD44 glycosylation or/and significant elongation of the receptor.

The adhesion assay was complemented by immunofluorescence analysis that confirmed this hypothesis. This analysis visualizes the CD44s expression/distribution and cell morphology (Fig. 4C,D). In agreement with flow cytometry results (MFI, Table 1), we can see that the CD44 expression (all CD44 isoforms) is much higher for the MKN45 (Fig. 4D) than for the AGS cell line (Fig. 4B). Another important difference that is visible from the immunofluorescence analysis is the tendency of the MKN45 to adhere in clusters, i.e. their adhesion is dependent on cell-cell contacts. This behavior is important especially in the case of the surface presenting HA of 1500 kDa, where we observed very few adherent cells/clusters and thus, we were not able to conclude on the influence of the CD44 blocking on the MKN45 cell adhesion to these substrates. Last, but not least important remark is that CD44 in AGS is detected mainly in the cytoplasm, while MKN45 express it at the cell membrane (green staining in Fig. 4C vs 4D). This different expression may lead to a higher sensitivity of the MKN45 cells towards the surface immobilized HA.

### Invadopodia formation by CD44 downstream activation

**Figure 5:**
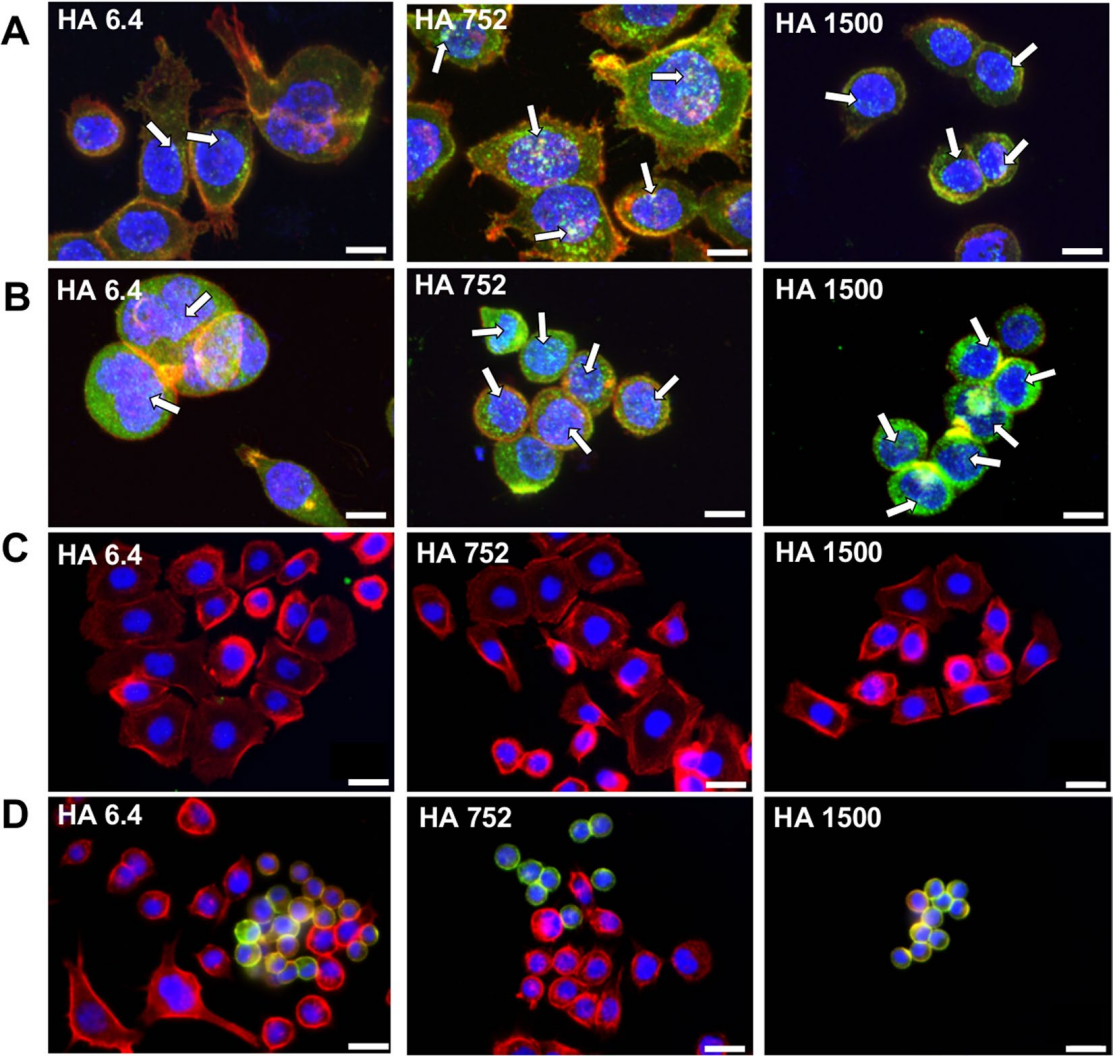
Expression of cortactin (**A**,**B**) and E-cadherin (**C**,**D**) by AGS (**A**,**C**) and MKN45 (**B**,**D**) cell lines. Cortactin, E-cadherin: green; actin: red; and nuclei: blue. Co-localization of actin/cortactin (**A**,**B**; white arrows) and actin/E-cadherin (**C**,**D**): yellow. Bars correspond to 10 µm for (**A**,**B**) and 20 µm for (**C**,**D**).

Cancer invasion and metastasis is linked to the existence of specific actin-rich membrane protrusions called invadopodia^47,48^. Several proteins are involved in the formation and development of these protrusions: Tks5, cortactin and neural Wiskott-Aldrich syndrome protein (N-WASP) compose the core structure of invadopodia^49,50^. Among these, cortactin is overexpressed in several cancer cells, where it regulates the secretion of matrix metalloproteinases (MMPs) to the stubs of degrading invadopodia, being crucial for the formation and function of these protrusions^51,52^. CD44-HA crosstalk is also involved in this process: it induces stabilization and maturation of invadopodia through the activation of 𝛼5𝛽1 integrin and phosphorylation of cortactin^53^. We therefore evaluated the formation of invadopodia and expression of cortactin by AGS and MKN45 cells cultured on the surfaces presenting the HA of different M_w_s (Fig. 5). Generally, MKN45 cells express more cortactin than AGS regardless of the used substrate (Fig. 5A vs B). The expression was affected by the M_w_: HA with higher M_w_ stimulate cortactin expression in both AGS (higher expression on HA of 752 kDa) and MKN45 cells (6.4 kDa ≪ 752 kDa < 1500 kDa). Cortactin co-localizes with actin at the cell membrane, which is associated with cortactin recruitment to cell–cell adhesive contacts in response to homophilic cadherin ligation^54^. This co-localization is well pronounced for MKN45 cells when HA of higher M_w_ is used on the substrates (Fig. 5B, yellow dots) and associated with the presence of cell clusters. In fact, these results are in good agreement with the E-cadherin expression observed for the MKN45 cells at the same type of cell-cell contacts (Fig. 5D). Cortactin also co-localizes with actin in punctate structures situated in the ventral surface of the cell, below its nucleus (Fig. 5A,B yellow staining shown by the white arrows). This co-localization together with the observed morphology and positioning of the structures is consistent with the formation of invadopodia^55,56^.

Invadopodia formation can be related with mechanical properties of the substrate^55,57^. In fact, the LbLs developed by us have different stiffness (Fig. 1D) that could trigger this process. LbL built-up from HA with lowest M_w_ (6.4 kDa) has highest surface stiffness. However, cells in contact with these substrates exhibit lower expression of cortactin/number of invadopodia, which is in contradiction with previous studies reporting higher surface stiffness as a promotor of invadopodia formation. We, therefore, conclude that the M_w_ of the HA is the key regulator of the invadopodia formation for the studied substrates. These results are in agreement with the observed expression of CD44 on the cell membrane: CD44 activates Src-kinase, which regulates the phosphorylation of cortactin and consequently, the formation of invadopodia^58^.

**Figure 6:**
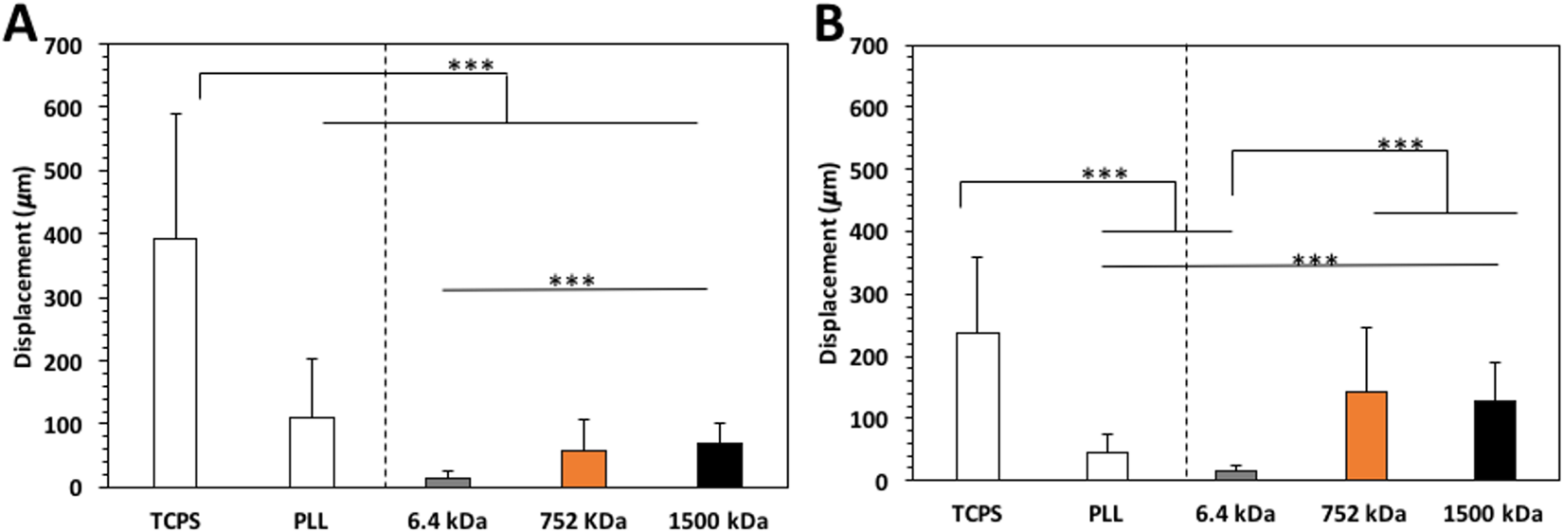
Single cell motility assay for AGS (**A**) and MKN45 (**B**) during 60 min of incubation. Significant differences (t-test) are marked with *** for a *p* < 0.001.

The M_w_ of the HA affected also the individual cell migration (Fig. 6A,B). Lowest cell motility and the shorter cell tracks (Supplementary Fig. S5) were observed for cells in contact with HA of 6.4 kDa. The migration ability of both AGS and MKN45 cells increased on substrates with HA of higher M_w_. Moreover, MKN45 cells present lower displacements than AGS on the control substrates (TCPS and PLL, i.e. no HA) but this behavior was switched on surfaces presenting HA of higher M_w_s (752 and 1500 kDa), where MKN45 cells (high surface expression of CD44) exhibit a higher cell motility than AGS cells.

## Conclusions

We demonstrate that immobilized HA have different effect on cancer cell behavior as compared with soluble HA supplemented with the culture media. The effect of HA depends not only on its presentation but also on the cancer cell type and the expression of CD44. We observed a relationship between the cell behavior and the M_w_ of the immobilized HA for cells that have high surface expression of CD44. Invasiveness properties such as low cell adhesion, high cell motility, high expression of cortactin, formation of invadopodia and cell clusters were enhanced for such cells when they are in contact with substrates presenting HA of high M_w_. While our results demonstrate that HA M_w_ influences cancer cell behavior, it also indicate that the way HA is presented (soluble vs immobilized) is of relevance for its biological functions. The use of platforms of immobilized HA resembling its presentation in the ECM (similar to the ones that we describe) to study cancer cell biology are probably more adequate than the use of soluble HA^59,60^. These platforms can be used to screen the activity of drugs on cancer cell invasion (whose evolution is dependent on the interaction between CD44-HA), leading to the achievement of consistent data that supports the further evaluation of selected drugs in the pipeline that leads to their use in the clinical practice.

## Materials and Methods

Unless otherwise stated, chemicals were used without further purification. Poly-L-lysine hydrobromide (PLL; M_w_ 30–70 kDa), N-(3-dimethylaminopropyl)-N′-ethylcarbodiimide hydrochloride (EDC) ≥ 98.0% and N-hydroxysuccinimide (NHS) were obtained from Sigma-Aldrich. Hyaluronic acid (HA; M_w_ 6.4 kDa, 752 kDa and 1500 kDa) was purchased from Lifecore. We used Anti-CD44 antibody [KM201] (ab25340) and Mouse IgG1 Kappa [MOPC-21]-Isotype 1 (ab18443) from Abcam for the CD44 blocking. Recombinant Human CD44 His tag protein was purchased from Biorbyt. Phalloidin–tetramethylrhodamine B isothiocyanate (phalloidin–TRITC), 4,6-diamidino-2-phenyindole, dilactate (DAPI) from Sigma-Aldrich and the monoclonal antibody to CD44 from Acris-Antibodies were used for immunostaining. The secondary antibody, Alexa Fluor 488 Rabbit Anti-Mouse IgG, was obtained from Invitrogen. The primary antibody Cortactin (H-191), sc-11408, was obtained from Santa Cruz Biotechnology.

### Formation and characterization of the PLL-HA bilayer system

The substrates used in this study were TCPS coverslips (13 mm diameter). The bilayer deposition was performed as previously described^28^. Briefly, the coverslips were immersed into a polycation solution (PLL, 0.5 mg/mL in 0.15 M NaCl) for 15 min, then removed and washed (0.15 M NaCl, pH ≈ 6.0–6.5) and then immersed into the polyanion solution (HA, 1 mg/mL in 0.15 M NaCl) supplemented with a crosslinking agent (EDC, 400 mM and NHS, 100 mM) for 15 min. The coated TCPS (TCPS-PLL-HA_m_, where m represents the M_w_ of the HA) were rinsed with water to remove the excess of salt, dried at room temperature and characterized or used in further studies.

### ζ-potential measurements

SurPASS electrokinetic analyzer (Anton Paar, Graz, Austria), was used to determine the zeta potentials of the assembled films. The TCPS-PLL-HAm were mounted in an adjustable disk gap cell (14 mm diameter). The gap between the discs was adjusted to approx. 110 μm and an electrolyte (1 mM NaCl, pressure of 400 mbar) was flowed through the cell. The streaming currents (I_str_) were determined at different pH values within the range 5.5 to 10 automatically adjusted by the addition of NaOH (0.05 M) to the solution. The respective ζ-potentials were calculated and averaged over three measurements using Attract 2.0 software (this software uses Smoluchowski equation for calculation of ζ-potentials from I_str_).

### Atomic Force Measurement (AFM)

experiments were performed on a JPK NanoWizard 3 (JPK, Germany). The roughness and mechanical properties of the films were measured under QI Advanced Mode using SNL-10 probes (resonance frequency of ~65 kHz; spring constant of ~0.34 N/m; Bruker, Germany) with the exception of the PLL coated surface where a TAP525 probe (resonance frequency of ~525 kHz; spring constant of ~200 N/m, Bruker, Germany) was used. The Young modulus of the films were calculated by fitting the approach curves with the Hertz model, using a paraboloid tip shape (applying a very short indentation range, 0.5–1 nm – to eliminate contribution of the underlying substrate). All the probes were calibrated by contact with the underlying surfaces, from which sensitivity was determined, and by fitting the cantilever resonance frequency. All the experiments were performed in air, while the surfaces were humidified using a drop of water.

### Quartz Crystal Microbalance (QCM-D) *in situ* measurements and modeling

The substrates used for this characterization were gold-coated AT-cut quartz crystals (QSX301, Q-Sense, Sweden). The crystals were washed several times with ethanol, dried under N_2_, and immediately placed in the QCM-D flow chamber (E4 instrument, Q-Sense). A stable baseline was acquired in 0.15 M solution of NaCl at 25 °C. The PLL solution (0.5 mg/mL in 0.15 M NaCl) was added to the chamber under a flow rate of 50 μL/min for 15 minutes. The sensor was rinsed with 0.15 M NaCl aqueous solution to remove loosely bound material and HA solution (1 mg/mL in 0.15 M NaCl) supplemented with the crosslinking agents 4 EDC:1 NHS was injected (50 μL/min) in the flow chamber for 15 min. After rinsing (0.15 M NaCl), Human CD44 His-tag protein (2 μg/mL in 1% BSA in phosphate-buffered saline (PBS)) was flowed onto the deposited PLL-HA bilayer until signal stabilization. Finally, the sensor was rinsed (0.15 M NaCl) to remove loosely bound protein. All measurements were performed at several harmonics (n = 3, 5, 7, 9 and 11). ΔF/n and ΔD were fitted for the 5^th^, 7^th^ and 9^th^ overtone using the Q-Tools software (v 3.0.6.213). Voigt element-based model was applied to obtain the thickness of the PLL-HA bilayers. The mass of the wet films and of the adsorbed CD44, was calculated by fitting the data in an interactive fashion, and by assuming a fluid density of 1020 kg/m^3^, a fluid viscosity of 0.001 kg/m.s, and a layer density of 1200 kg/m^3^.

### Culture, expansion and seeding of gastric cancer cell lines

Human gastric cancer cell lines AGS and MKN45 were cultured in RPMI-1640 medium (Sigma-Aldrich) supplemented with 10% heat-inactivated fetal bovine serum (FBS; Biochrom AG, Germany) and 1% antibiotic/antimycotic solution (final concentration of penicillin 100 units/mL and streptomycin 100 mg/mL; Gibco, UK). Cells were cultured in a 5% CO_2_ incubator at 37 °C.

Before the *in vitro* studies, the substrates (TCPS-PLL-HAm) were sterilized by exposing them to UV light for 30 min. Confluent AGS and MKN45, were harvested and seeded on TCPS-PLL-HA_m_ at a density of 11000 cells/cm^2^. The cells were cultured for 3 days. Uncoated TCPS substrates were processed as the other samples and used as control.

### Protein expression

After each time point the samples were washed twice with PBS, fixed in 10% neutral buffered formalin for 30 min at 4 °C, permeabilized with 0.2% Triton X-100 in PBS for 5 min, and blocked with 3% BSA in PBS for 30 min at room temperature. The CD44 expression was evaluated by CD44 monoclonal antibody (clone 8E2F3, 1:400 in 1% w/v BSA/PBS), followed by rabbit anti-mouse Alexafluor-488 (1:500 in 1% w/v BSA/PBS). Cortactin was visualized by using cortactin rabbit polyclonal antibody (H-191, 1:400), followed by the secondary rabbit anti-mouse Alexafluor-488 (1:500 in 1% w/v BSA/PBS). E-Cadherin was stained using rabbit monoclonal antibody (EP700Y, 1:500), followed by the secondary rabbit anti-mouse Alexafluor-488 (1:500 in 1% w/v BSA/PBS). The cells were observed under confocal laser scanning microscope (TCS SP8, Leica, Germany).

### Morphology, distribution and motility of AGS and MKN45

A phalloidin−TRITC conjugate was used (1:200 in PBS for 30 min) to assess cytoskeleton organization and cell morphology of the studied cell lines. Nuclei were counter-stained with DAPI (1 mg/mL in 1% BSA in PBS for 30 min). Samples were washed with PBS, mounted with Vectashield (Vector) on glass slides and observed under an Imager Z1 microscope (Zeiss, Germany) and photographed using an Axio Cam MRm camera (Zeiss, Germany) to evaluate the number of adherent cells. Confocal laser scanning microscope (TCS SP8, Leica, Germany) was used to evaluate the morphology of adherent cells. Live monitoring of individual cell motility was performed at 37 °C in an inverted microscope (Zeiss Axio Observer) equipped with a temperature and CO_2_ control device (5% CO_2_). Time-lapse images (40X) were captured every 1 min using Zen software. Cells were continuously observed for 60 min. Image stacks were analyzed with the image processing software Fiji (http://fiji.sc/wiki/index.php/Fiji) and cell displacement quantified using Track Mate Plugin.

### Flow Cytometry analysis

AGS (P8) and MKN45 (P7) were detached with TrypLe. TrypLe is a mix of recombinant cell-dissociation enzymes. Its action is milder than the commonly used porcine trypsin: TrypLe preserves better cell-surface epitope expression when compared to trypsin^61^. Detached cells were re-suspended in PBS and incubated for 20 min at room temperature with mouse anti-human CD44-PE antibody (clone 515) and mouse anti-human CD44v6 monoclonal antibody (MA54), following manufacturer-recommended concentrations. Cells were subsequently washed with PBS, centrifuged, fixed in 1% paraformaldehyde (Sigma), and analyzed on a FACs Calibur Flow Cytometer (BD Biosciences) using the Flowing Software v2.5.1.

### Image analysis

The quantification of adherent cells and the time for adhesion were performed using ImageJ analysis software (Version 2.0.0-rc-34/1.50a).

### Statistical analysis

The normality of the data was evaluated using Shapiro−Wilk test (*p* < 0.05). When the data did not follow a normal distribution an initial Kruskal−Wallis test was executed followed by Mann-Whitney test. T-test was performed for data with a normal distribution. In all the cases, a significant variation was only considered for a ^***^p < 0.001.

## Electronic supplementary material

Supplementary Information

Movie 1 - AGS 6.4kDa

Movie 2 - AGS 752kDa

Movie 3 - AGS 1.5MDa

Movie 4 - AGS PLL

Movie 5 - AGS TCPS

Movie 6 - MKN45 6.4kDa

Movie 7 - MKN45 752kDa

Movie 8 - MKN45 1.5MDa

Movie 9 - MKN45 PLL

Movie 10 - MKN45 TCPS

## Data Availability

All data generated or analyzed during this study are included in this published article (and its Supplementary Information files).
