# Supplementary material for: Molecular weight of surface immobilized hyaluronic acid influences CD44-mediated binding of gastric cancer cells
Source: Sci Rep. 2018 Oct 30;8:16058. doi: 10.1038/s41598-018-34445-0 (PMC6207784; doi:10.1038/s41598-018-34445-0)
Supplement: Supplementary file 1 — Supplementary Information [file 41598_2018_34445_MOESM1_ESM.pdf]

Supplementary Information

Molecular weight of surface immobilized hyaluronic acid influences CD44-mediated binding of gastric cancer cells

Sara Amorim[1,2,3], Diana Soares da Costa[1,2], Daniela Freitas[4,5,6], Celso A. Reis[4,5,6,7], Rui L. Reis[1,2,3], Iva Pashkuleva[1,2,*] and Ricardo A. Pires[1,2,3,*]

[1] 3B's Research Group, I3Bs – Research Institute on Biomaterials, Biodegradables and Biomimetics, University of Minho, Headquarters of the European Institute of Excellence on Tissue Engineering and Regenerative Medicine, AvePark, Parque de Ciência e Tecnologia, Zona Industrial da Gandra, 4805-017 Barco, Guimarães, Portugal
[2] ICVS/3B's - PT Government Associate Laboratory, Braga/Guimarães, Portugal
[3] The Discoveries Centre for Regenerative and Precision Medicine, Headquarters at University of Minho, Avepark, 4805-017 Barco, Guimarães, Portugal
[4] Instituto de Investigação e Inovação em Saúde - i3S, Universidade do Porto, Portugal
[5] Institute of Molecular Pathology and Immunology of the University of Porto – IPATIMUP, Portugal
[6] Institute of Biomedical Sciences Abel Salazar, University of Porto, Porto, Portugal
[7] Department of Pathology and Oncology, Faculty of Medicine, Porto University, Porto, Portugal

E-mails: pashkuleva@dep.uminho.pt; rpires@dep.uminho.pt

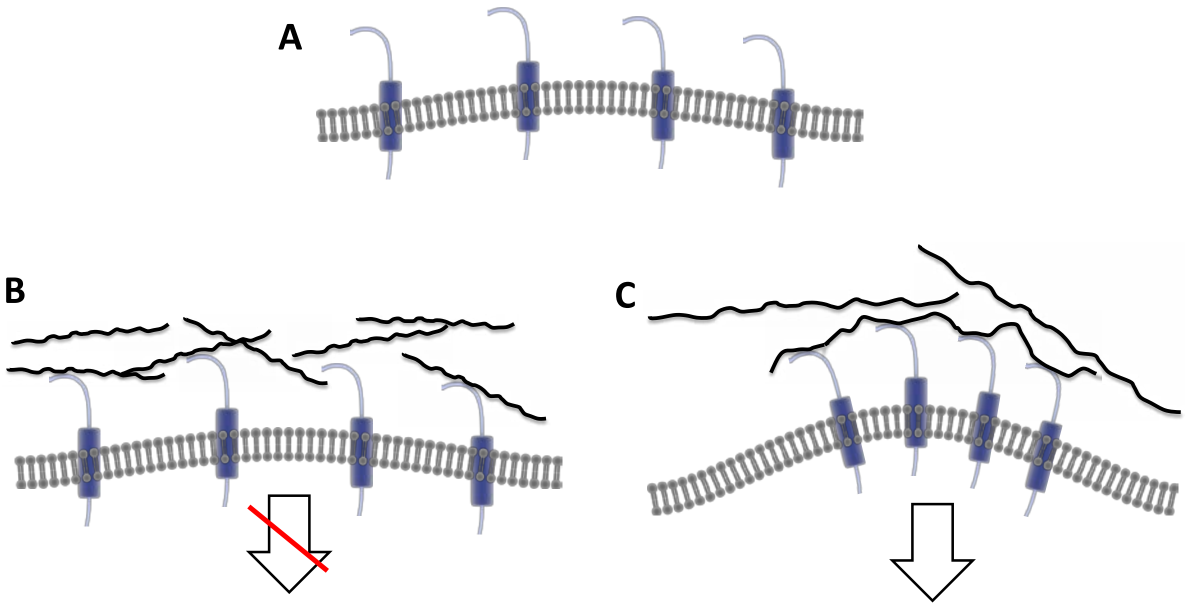

Figure S1. Schematic presentation of the interactions between the cell-surface glycoprotein CD44 (A) and hyaluronan in solution (B and C). Oligomers of hyaluronan bind monovalently to CD44 and do not activate the following intracellular signaling (B). In fact, they can replace the endogenous hyaluronan of high molecular weight and attenuate the signaling. On the contrary, hyaluronan of high molecular

weight bind CD44 multivalently resulting in CD44 clustering and activation in different downstream signaling.

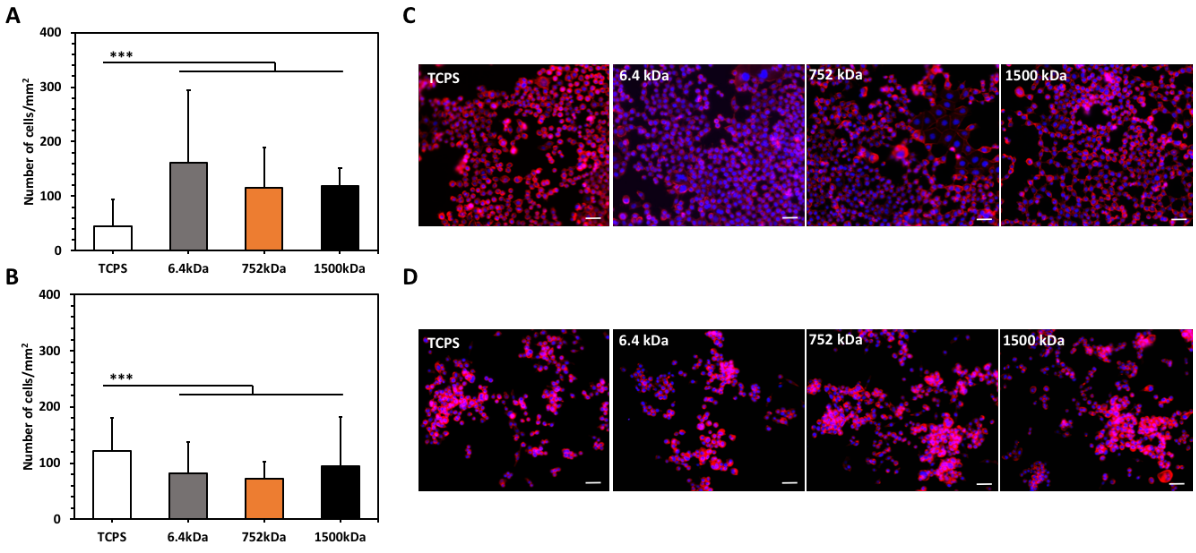

Figure S2. Number of adherent cells (AGS, A and MKN45, B) and morphology of AGS (C) and MKN45 (D) after 72h of culture on TCPS in the presence of hyaluronan with different M_ws supplemented to the culture media at concentration of 1 mg/mL.

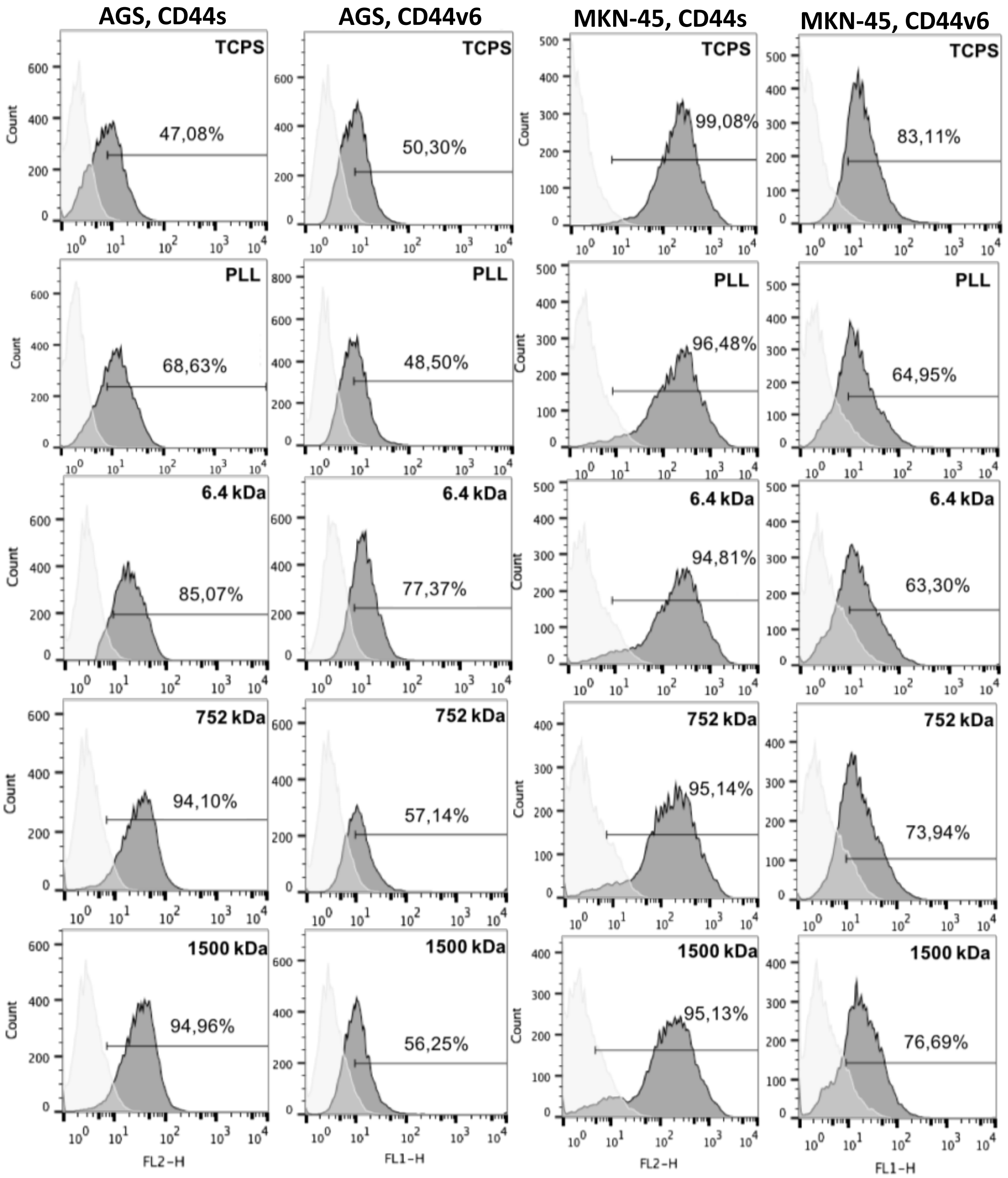

Figure S3. Flow cytometry analysis of surface marker CD44s and CD44v6 for the AGS and MKN-45, after 72h of cell culture in each surface.

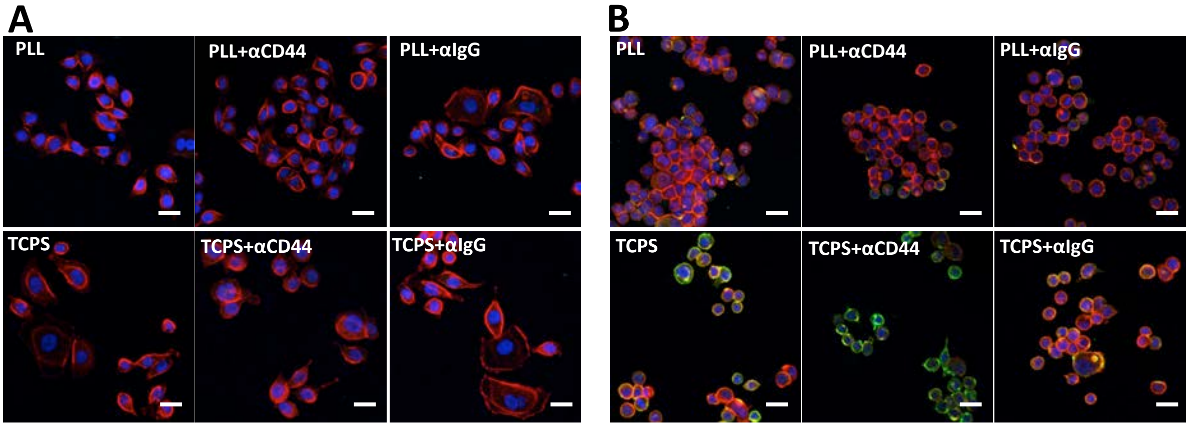

Figure S4. Immunohistochemistry of AGS (A) and MKN-45 (B) cultured on control surfaces (TCPS and PLL) during 72 h without or after CD44 blocking (green for CD44, red for actin and blue for nuclei). IgG was used as an isotype control. Bars correspond to 50 μm.

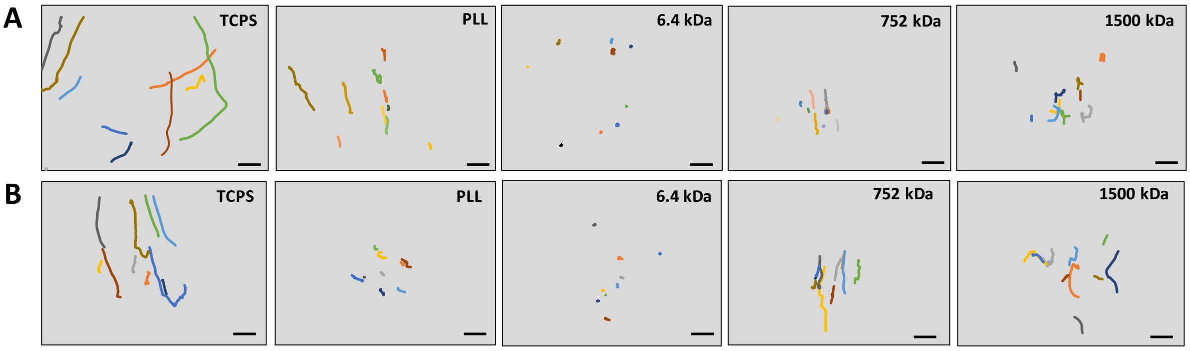

Figure S5. Representative example of single cell paths. Images show the cells tracking during 60 minutes of incubation, of AGS (A) and MKN-45 (B) cells on the different surfaces. The displacements of cells are represented as color lines. Each plot represents 1 individual cell track. Bars correspond to 100 μm.
